# Protocol for low-cost photographic digitization and automated HTML publication of envelope-preserved entomological museum collections

**DOI:** 10.1016/j.xpro.2026.104871

**Published:** 2026-09-28

**Authors:** Davide Tamagnini, Claudio Chimenti

**Affiliations:** 1 Department of Biology and Biotechnologies “Charles Darwin”, Sapienza University of Rome, 00185 Rome, Italy

**Keywords:** Developmental biology, Environmental sciences, Evolutionary biology

## Abstract

Here, we present a protocol for digitizing envelope-preserved entomological museum specimens. We describe steps for acquiring images, transcribing labels into Darwin Core records, and validating taxonomy and geography. We then detail procedures for linking images to metadata and automatically generating searchable specimen and taxon HTML pages for open-access publication. We demonstrate the workflow using *Odonata* specimens from the Zoological Museum of Sapienza University of Rome.

For complete details, please refer to Tamagnini et al.^1^

## Highlights

• Low-cost workflow for photographic digitization of entomological collections
• Standardized integration of specimen images and Darwin Core metadata
• Python scripts automate specimen-, species-, and identifier-based HTML pages
• Static web publication enables open access without a dedicated database server

## Abstract (editor highlights)

Publisher’s note: Undertaking any experimental protocol requires adherence to local institutional guidelines for laboratory safety and ethics.

## Graphical abstract

**Figure:**
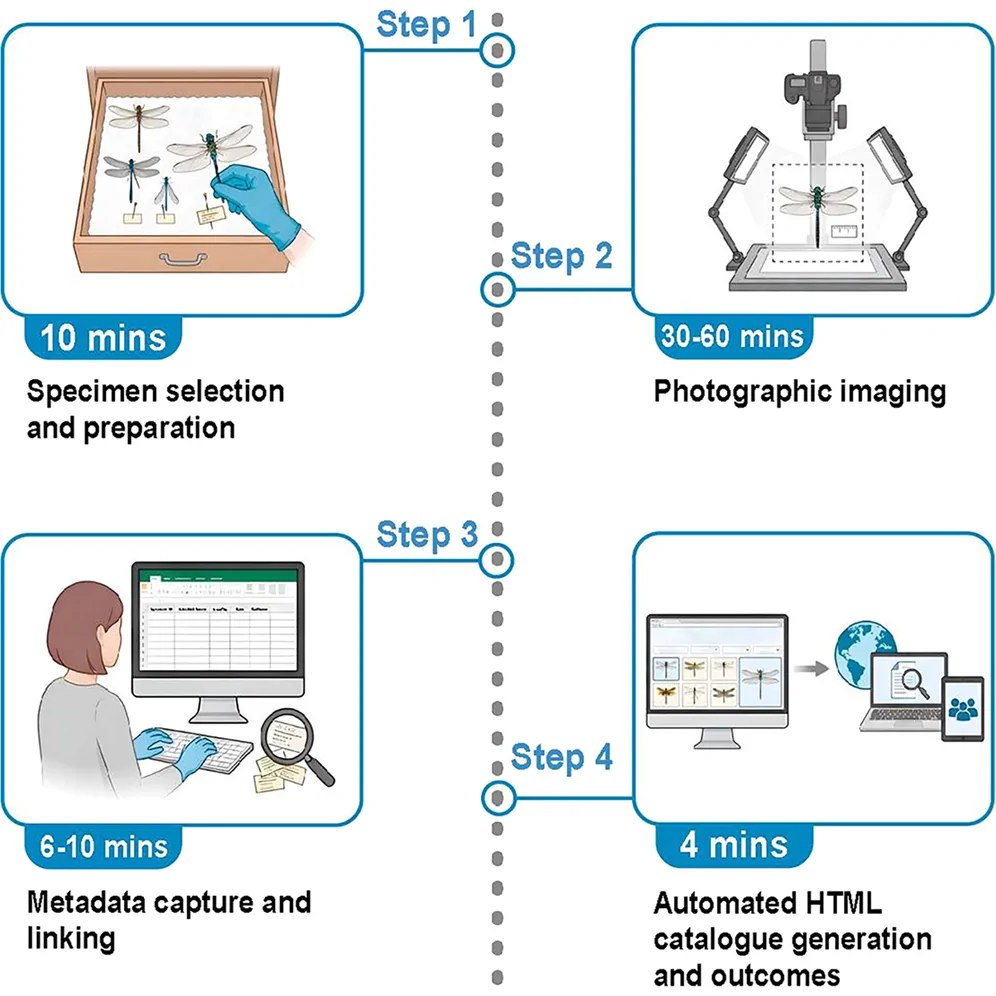

## Before you begin

### Digitization preparation

Natural history collections are critical research infrastructure, serving as biological archives that preserve essential temporal and spatial records of global past and present biodiversity.^2^^,^^3^ Entomological collections are among the largest museum collections and are vital for both traditional taxonomy and for detecting the impacts of climate change and human activity on ecosystems.^4^^,^^5^ Despite their enormous potential, much of the data held in these collections remain largely unexplored and underutilized due to slow traditional digitization processes.^6^

Digitization and the creation of open-access databases fitting FAIR principles (i.e., Findability, Accessibility, Interoperability, and Reusability^7^) are essential to prevent the loss of non-renewable information from catastrophic events or mismanagement.^8^^,^^9^ Furthermore, providing accessible, high-quality data through open-access platforms, like the Global Biodiversity Information Facility, accelerates biodiversity and evolutionary research (e.g., evolutionary patterns through time and space^9^^,^^10^) and informs conservation planning.^5^^,^^11^

Large-scale digitization of entomological collections provides critical infrastructure for biodiversity research, taxonomy, biogeography, conservation biology, and ecological monitoring.^12^ Open-access specimen datasets enable remote examination of voucher material, facilitate verification of identifications, improve access to historical occurrence records, and support integration with global biodiversity infrastructures such as GBIF. In the insect order Odonata, which includes dragonflies and damselflies, digitized collections are particularly valuable for documenting historical distributions, phenological change, and long-term responses to environmental change. The use of structured metadata and automated web publication increases both the accessibility and the long-term scientific usability of museum collections.

The protocol presented here provides a reproducible workflow for the standardized handling, imaging, data structuring, and online publication of dry specimens preserved in paper or card envelopes. To demonstrate the workflow, we use the historical Odonata collection of the Zoological Museum of Sapienza University of Rome as a case study. The physical imaging procedure is optimized for envelope-preserved odonates, whereas the identifier-management, data-structuring, image-linking, and HTML-generation components can be adapted to other collection types with preservation-specific modifications.

Previous digitization workflows have often focused primarily on individual stages, such as imaging, colour correction, three-dimensional specimen imaging, morphological digitization, or data publication.^13^^,^^14^ In contrast, the protocol presented here integrates specimen photography, stable identifier assignment, Darwin Core-based data management, taxonomic and geographic quality control, automated association of media and records, and generation of searchable static HTML interfaces within a single reproducible pipeline.

The protocol described here was developed and validated during the digitization of the historical Odonata collection of Sapienza University of Rome, comprising 2,406 catalogued records (Table 1).

**Table 1:** User-oriented subset of the complete Darwin Core-based occurrence dataset

occurrenceID	eventDate	associatedMedia	scientificName	identifiedBy	taxonRank	Continent	Country	decimalLongitude	decimalLatitude	Family	Genus
MZURODOB00030	01/11/1976	MZURODOB00030_specimen-lateral_01.jpg	Aeshna cyanea (Müller, 1764)	P. Nicolai	species	Europe	Italy	42.9533263462919	12.0093021467651	Aeshnidae	Aeshna
MZURODOB00031	01/11/1976	MZURODOB00031_specimen-lateral_01.jpg	Aeshna cyanea (Müller, 1764)	P. Nicolai	species	Europe	Italy	42.9533263462919	12.0093021467651	Aeshnidae	Aeshna
MZURODOB00032	01/11/1976	MZURODOB00032_specimen-lateral_01.jpg	Aeshna cyanea (Müller, 1764)	P. Nicolai	species	Europe	Italy	42.9533263462919	12.0093021467651	Aeshnidae	Aeshna
MZURODOB00033	17/09/1995	MZURODOB00033_specimen-lateral_01.jpg	Aeshna cyanea (Müller, 1764)	C. Utzeri	species	Europe	Italy	42.8747753243228	12.7366181168905	Aeshnidae	Aeshna
MZURODOB00034	17/09/1995	MZURODOB00034_specimen-lateral_01.jpg	Aeshna cyanea (Müller, 1764)	C. Utzeri	species	Europe	Italy	42.8747753243228	12.7366181168905	Aeshnidae	Aeshna
MZURODOB00035	17/09/1995	MZURODOB00035_specimen-lateral_01.jpg	Aeshna cyanea (Müller, 1764)	C. Utzeri	species	Europe	Italy	42.8747753243228	12.7366181168905	Aeshnidae	Aeshna
MZURODOB00036	07/10/1977	MZURODOB00036_specimen-lateral_01.jpg	Aeshna cyanea (Müller, 1764)	C. Utzeri	species	Europe	Italy	43.8286	11.103752	Aeshnidae	Aeshna
MZURODOB00037	22/07/1980	MZURODOB00037_specimen-lateral_01.jpg	Aeshna cyanea (Müller, 1764)	P. Nicolai	species	Europe	Italy	43.3316117275593	11.9241525455212	Aeshnidae	Aeshna
MZURODOB00038	12/09/1991	MZURODOB00038_specimen-lateral_01.jpg	Aeshna cyanea (Müller, 1764)	M. Rampini, M. Cobolli	species	Europe	Belgium	50.5865965358111	5.57072851451592	Aeshnidae	Aeshna
MZURODOB00039	02/10/1986	MZURODOB00039_specimen-lateral_01.jpg	Aeshna cyanea (Müller, 1764)	G. Carchini, C. Utzeri	species	Europe	France	41.7343051750342	9.33510201837415	Aeshnidae	Aeshna
MZURODOB00040	20/08/1949	MZURODOB00040_specimen-lateral_01.jpg	Aeshna cyanea (Müller, 1764)	P. Bisleti	species	Europe	Switzerland	46.4942537167126	7.67009436063212	Aeshnidae	Aeshna
MZURODOB00041	23/08/1951	MZURODOB00041_specimen-lateral_01.jpg	Aeshna mixta Latreille, 1805	C. Consiglio	species	Europe	Nederlands	52.078854439634	5.83246774025048	Aeshnidae	Aeshna
MZURODOB00042	23/08/1951	MZURODOB00042_specimen-lateral_01.jpg	Aeshna mixta Latreille, 1805	C. Consiglio	species	Europe	Nederlands	52.078854439634	5.83246774025048	Aeshnidae	Aeshna
MZURODOB00043	05/10/1971	MZURODOB00043_specimen-lateral_01.jpg	Aeshna mixta Latreille, 1805	C. Consiglio	species	Europe	Italy	41.7430813868143	12.4011379947984	Aeshnidae	Aeshna
MZURODOB00044	05/10/1971	MZURODOB00044_specimen-lateral_01.jpg	Aeshna mixta Latreille, 1805	C. Consiglio	species	Europe	Italy	41.7430813868143	12.4011379947984	Aeshnidae	Aeshna
MZURODOB00045	25/09/1950	MZURODOB00045_specimen-lateral_01.jpg	Aeshna mixta Latreille, 1805	C. Consiglio	species	Europe	Italy	41.7244864536972	12.6726742287293	Aeshnidae	Aeshna
MZURODOB00046	01/10/1948	MZURODOB00046_specimen-lateral_01.jpg	Aeshna mixta Latreille, 1805	M. Comba	species	Europe	Italy	41.8930880999585	12.4831161655412	Aeshnidae	Aeshna
MZURODOB00047	31/10/1947	MZURODOB00047_specimen-lateral_01.jpg	Aeshna mixta Latreille, 1805	G. Saracchi	species	Europe	Italy	41.8758317471991	12.2122809990536	Aeshnidae	Aeshna
MZURODOB00048	15/06/1973	MZURODOB00048_specimen-lateral_01.jpg	Aeshna isoceles (Müller, 1767)	W. Rossi	species	Europe	Italy	41.6072273963193	12.5098376606247	Aeshnidae	Aeshna
MZURODOB00049	15/06/1973	MZURODOB00049_specimen-lateral_01.jpg	Aeshna isoceles (Müller, 1767)	W. Rossi	species	Europe	Italy	41.6072273963193	12.5098376606247	Aeshnidae	Aeshna
MZURODOB00050	15/06/1973	MZURODOB00050_specimen-lateral_01.jpg	Aeshna isoceles (Müller, 1767)	W. Rossi	species	Europe	Italy	41.6072273963193	12.5098376606247	Aeshnidae	Aeshna
MZURODOB00051	15/06/1973	MZURODOB00051_specimen-lateral_01.jpg	Aeshna affinis Vander Linden, 1820	W. Rossi	species	Europe	Italy	41.6072273963193	12.5098376606247	Aeshnidae	Aeshna
MZURODOB00052	07/08/1975	MZURODOB00052_specimen-lateral_01.jpg	Aeshna affinis Vander Linden, 1820	C. Utzeri	species	Europe	Italy	41.7430813868143	12.4011379947984	Aeshnidae	Aeshna
MZURODOB00053	11/07/1975	MZURODOB00053_specimen-lateral_01.jpg	Aeshna affinis Vander Linden, 1820	C. Utzeri	species	Europe	Italy	41.7430813868143	12.4011379947984	Aeshnidae	Aeshna

The 12 fields shown here illustrate the principal values used by the Python workflow to associate specimen images with records and generate searchable HTML interfaces. The underlying dataset contains additional terms covering institutional provenance, catalogue information, taxonomic hierarchy and authorship, collecting event, geography, georeferencing, specimen attributes, and associated media. Twenty-four records are shown as examples; the complete dataset of 2,406 records is provided as Data S1 in Excel format.

### Innovation

The innovation of this protocol lies in the integration of widely available digitization components into a low-cost, end-to-end pipeline. Rather than presenting photography, spreadsheet transcription, Darwin Core, or web hosting as individually novel, the workflow connects them directly from specimen preparation to public dissemination. It covers stable specimen identification, high-resolution imaging, label transcription, structured metadata capture, validation, image-record linkage, automated HTML generation, and open-access sharing through an easy-to-read static website.

Custom Python scripts are the central automation layer of the workflow. They read the curated specimen dataset, associate images with records through stable specimen identifiers, and generate interconnected specimen-level and taxon-level HTML pages. The resulting interface supports consultation by multiple fields, including accepted scientific name, identifier, locality, collecting date, catalogue number, and associated image. Because the output consists of static HTML files, it can be deployed through GitHub Pages or a similar service without a database server. The use of Darwin Core terms further supports data exchange and reuse, while the public interface intentionally displays a concise user-oriented subset of the complete underlying record. Taxonomic validation and name normalization are performed before page generation so that spelling variants, synonyms, obsolete combinations, and species-versus subspecies-level identifications do not create misleading duplicate taxon pages.

### Institutional permissions

No experiments on live animals were conducted for this protocol. All specimens used in this study belong to the historical Odonata collection housed at the Zoological Museum of Sapienza University of Rome (MZUR). Specimen handling and digitization were conducted in accordance with the museum’s collection-management procedures.

### Specimen preparation and imaging system

**Timing: 10 min**

This section describes workspace preparation, imaging-system setup, and preparation of data-capture materials before specimen digitization.

1. Workspace and specimen preparation.
  a. Ensure that the workspace can safely accommodate specimen drawers, envelopes, imaging equipment, and temporary staging materials, while allowing specimens to be handled without crowding or obstruction.
  b. Before or during the first imaging step, verify or assign each specimen a stable, unique identifier.
    ***Note:*** In the case study, identifiers combine the institutional prefix, collection code, and a sequential specimen number (e.g., MZURODOB00001).
  c. Ensure that labels remain legible and securely associated with the correct specimen. Use acid-free archival labels and permanent pigment-based archival ink; standard water-soluble or non-archival inks should not be used.
2. Imaging system setup (Figure 1).
  a. Mount and align the specimen stage, reference elements, lighting system, and computer before powering on and configuring the equipment.
  b. Mount the camera on a stable tripod or, preferably, a fixed vertical copy stand. Position the optical axis perpendicular to the imaging surface to minimize perspective distortion and to maintain reproducible framing and focus across specimens.
  c. Place a positioning frame or template on the imaging surface to ensure consistent placement, scale, orientation, and cropping of the specimen card, label, and identifier.
  d. Align two diffuse LED light sources symmetrically at approximately 45° relative to the imaging surface, one on either side of the specimen, to reduce shadows, glare, and uneven illumination.^15^
  e. Power on the equipment and calibrate image resolution, white balance, aperture, exposure time, focus, and scale before specimen acquisition. A digital camera with at least 20 megapixels is recommended. Capture and verify a calibration image containing a metric scale and, where available, a colour reference. Repeat calibration whenever camera height, magnification, lighting, or exposure settings change.

**Figure 1:**
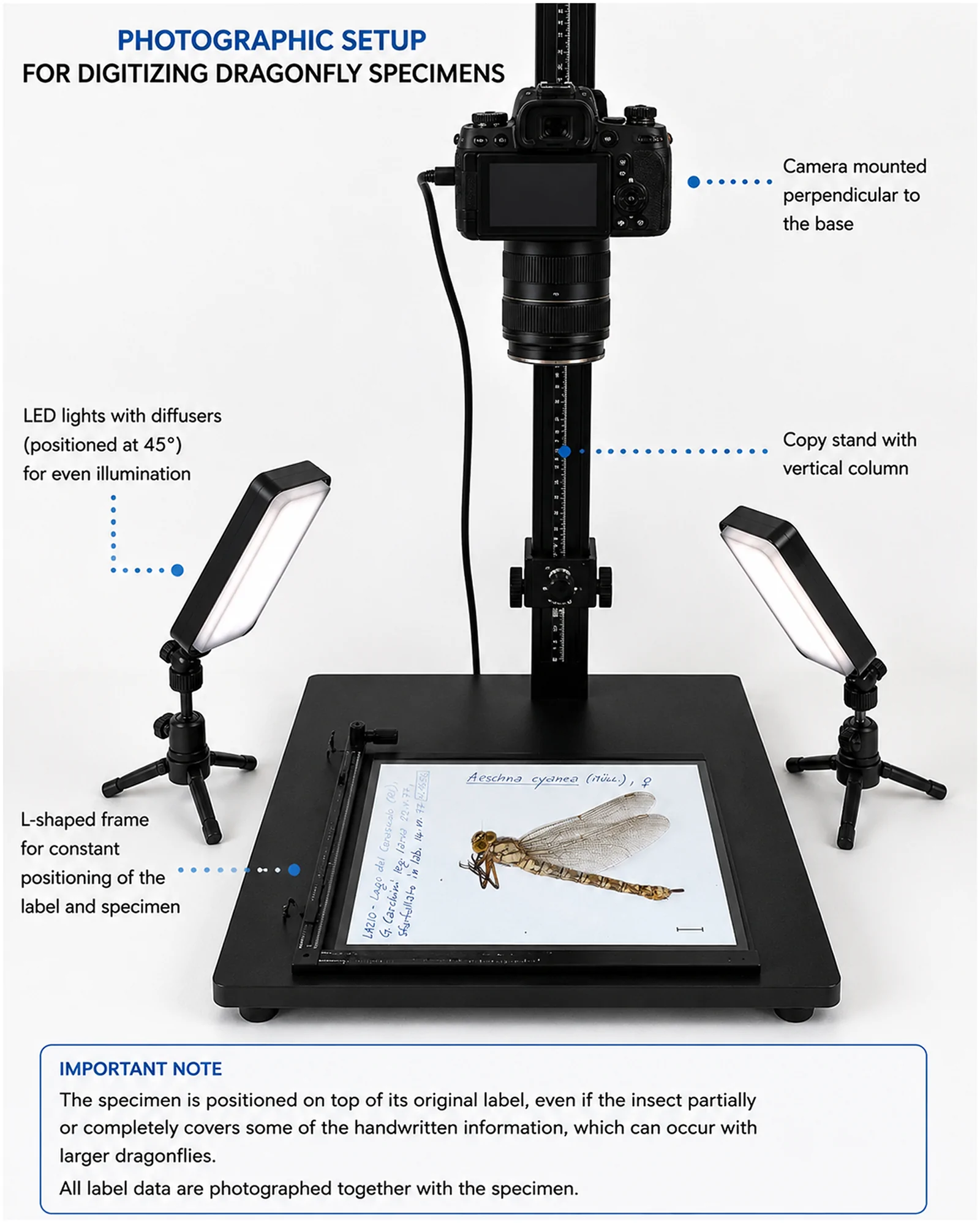
Example of photographic assembly station used in this protocol, showing camera positioning, lighting configuration, specimen stage, and background frame

**CRITICAL:** Complete and verify colour, exposure, focus, and scale calibration before imaging specimens. Maintain constant camera-to-stage distance and lighting throughout each calibrated imaging batch.

3. Data-capture and workflow materials.
  a. Use spreadsheet software capable of reading and exporting.xlsx or UTF-8 encoded.csv files to record and validate specimen data.
  b. Prepare the complete Darwin Core-based metadata template before data entry. The underlying dataset should include stable identifiers, institutional and collection information, taxonomy, collecting event, geography, georeferencing, identification, and associated media.
    ***Note:*** The public HTML interface may display a smaller user-oriented subset of the fields included in the complete underlying dataset.
  c. Ensure that all workflow materials, including staging trays, imaging mounts, rulers for scale bars, and specimen handling tools, are prepared before image acquisition.
  d. Confirm that local and external backup storage systems are available and functioning properly prior to data collection and image processing.
    ***Note:*** Redundant data storage minimizes the risk of data loss during large-scale digitization workflows.

## Key resources table

**Table:** 

REAGENT or RESOURCE	SOURCE	IDENTIFIER
**Experimental models: Organisms/strains**		

Dried Odonata collection	Zoological Museum of Sapienza University of Rome (MZUR)	https://doi.org/10.15468/hhq5g5

**Software and algorithms**		

Python 3.x	Python Software Foundation	https://www.python.org
Excel	Microsoft Corporation	Microsoft 365
Photoshop	Adobe	CS6
Darwin Core metadata template	This study	Occurrence_Odonata_GBIF_Template_DTCC_PUBBLICATO_REVISIONE_07_2026.xlsx
Image filename extraction script	GitHub repository	Odonata-digitization-scripts/elenca_file_1.py at main · Sapienza-BBCD/Odonata-digitization-scripts
Home page builder script	GitHub repository	Odonata-digitization-scripts/Dragonfly_01a_VER_07_2026.py at main · Sapienza-BBCD/Odonata-digitization-scripts
HTML specimen page generator script	GitHub repository	Odonata-digitization-scripts/Dragonfly_02_VER_07_2026.py at main · Sapienza-BBCD/Odonata-digitization-scripts
HTML GBIF code reconstruction interface script	GitHub repository	Odonata-digitization-scripts/Dragonfly_03_VER_07_2026.py at main · Sapienza-BBCD/Odonata-digitization-scripts
Species summary page generator script	GitHub repository	Odonata-digitization-scripts/Dragonfly_04_VER_07_2026.py at main · Sapienza-BBCD/Odonata-digitization-scripts
Identifier summary script	GitHub repository	Odonata-digitization-scripts/Dragonfly_05_VER_07_2026.py at main · Sapienza-BBCD/Odonata-digitization-scripts
Workflow on GitHub	GitHub repository	Odonata Collection

**Other**		

Digital camera	Nikon Corporation	Model Nikon Z7
Macro lens	Nikon Corporation	Model NIKKOR MC 105/2.8 S
LED lighting system	Equivalent commercial or custom-built system	Two dimmable diffuse LED panels, approximately 5000–5600 K; high CRI recommended
Specimen staging frame	Custom-built	N/A

## Step-by-step method details

### Preliminary steps for photographic sampling

**Timing: 30–60 min per 20 specimens**

This section describes specimen selection and staging before image acquisition.

1. Specimen selection.
  a. Select dry specimens preserved in paper or card envelopes and verify the presence, physical association, and readability of all accompanying labels.
  b. Record information that cannot be reliably reconstructed from the photograph alone, including specimen-label association, number of individuals, detached parts, storage context, and condition.
    ***Note:*** Information visible on labels may be transcribed after imaging, but should be checked against the physical material when ambiguity exists.
  c. Do not exclude specimens solely because they are damaged, incomplete, or unidentified to species. Document their condition and the lowest reliable identification level in the dataset. Postpone imaging only when handling would pose an unacceptable risk to the specimen or associated labels.
    ***Note:*** Damaged or incomplete specimens can provide important collection-management and historical information and should normally remain represented in the digital archive.
    ***Note:*** The physical imaging procedure is optimized for dry Odonata specimens stored in paper or card envelopes, typically 30–80 mm in body length. Pinned, slide-mounted, or fluid-preserved specimens require different staging and imaging procedures, although the data-management and HTML-generation components can be easily adapted from the present protocol.
2. Specimen staging (Figure 2).
  a. Position the envelope, card, or specimen according to a predefined orientation appropriate to the preservation method and taxonomic group.
    ***Note:*** In the present case study, envelope-preserved odonates are imaged laterally when this reflects their storage position and best displays diagnostic structures. Acquire additional dorsal, ventral, or detail views when required.
  b. Using fine forceps, center the specimen and maintain the same primary orientation across comparable records. Keep the original label and stable identifier within or directly associated with the imaging frame.
  c. Adjust lighting to minimize shadows and reflections.
  d. Verify focus, white balance, scale placement, identifier visibility, and framing before each imaging batch and whenever the setup changes.
    **Pause point:** Prepared specimens may remain positioned on the imaging stage for up to 2 h before photography, provided they are protected from excessive light exposure, dust, vibrations, and accidental displacement. Longer staging periods are not recommended, as specimen or label movement may reduce image consistency and alignment.
    ***Note:*** Apply a consistent filename convention from the first image onward. Use the stable specimen identifier as the filename prefix rather than a taxon name, because taxonomic identifications may change over time. Recommended pattern: <occurrenceID>_<viewType>_<sequence>.<extension>, for example MZURODOB00001_specimen-lateral_01.tif.
    **CRITICAL:** Manipulate specimens only using fine entomological tweezers or forceps. Direct handling with fingers should be avoided to prevent accidental damage to fragile diagnostic structures.

**Figure 2:**
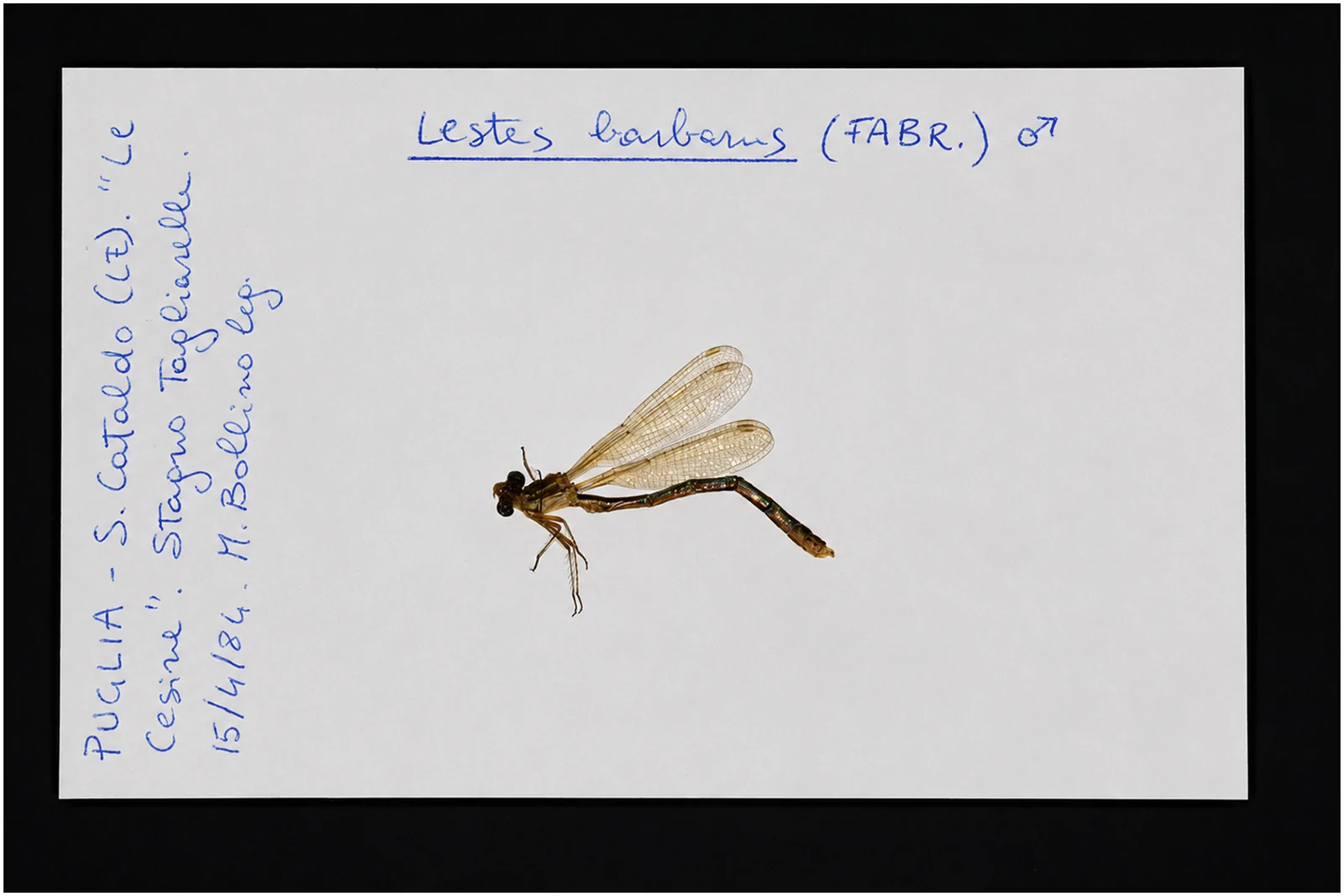
Lateral staging of an envelope-preserved odonate specimen within the imaging frame The original card and label remain associated with the specimen, which is positioned laterally at the centre of the frame with the head consistently oriented to the same side. Labels used for permanent collection management should be made of archival-quality paper and marked with permanent pigment-based archival ink.

### Specimen identification, high-resolution imaging, and postprocessing

**Timing: 6–10 min per specimen**

This section describes specimen identifier verification, high-resolution image acquisition, and standardized image postprocessing.

3. Verify or assign the specimen identifier.
  a. Retrieve the entomological envelope containing the specimen and all associated labels, and verify that these elements remain together throughout the workflow.
  b. Verify or assign a stable identifier consisting of the institutional prefix, collection code, and sequential specimen number. Use this identifier as the basis for filenames, metadata linkage, and the occurrenceID.

***Note:*** A barcode may be used where infrastructure permits, but a clearly printed human-readable identifier is sufficient when applied consistently.

**CRITICAL:** Never separate a specimen, its labels, its identifier, and its image log during staging or capture. Loss of this association compromises record traceability.

4. High-resolution image capture.
  a. Confirm uniform illumination, focus, exposure, colour balance, scale, and identifier visibility using a test image before beginning each batch.
  b. Capture images at the maximum practical native resolution of the camera using a macro lens and stable support.
  c. Retain original RAW files when available; generate a lossless preservation master (e.g., TIFF) and a high-quality JPEG derivative for web display.
  d. Record the camera model, pixel dimensions, colour profile, and relevant processing settings.
  e. Use tethered capture to a computer workstation when supported by the camera, to enable immediate quality control, identifier-based naming, and direct storage.

***Note:*** If tethered capture is unavailable, transfer files after each batch and verify completeness before returning specimens to storage.

5. Image postprocessing.
  a. Transfer images to the workstation or save them directly by tethered capture. Maintain at least two verified storage copies and preserve the original unedited files.
  b. Establish the correct orientation during capture whenever possible. If rotation is required, use non-destructive image-processing software and preserve the original master file.
    ***Note:*** Adobe Photoshop was used in the case study, but equivalent software may be substituted.
    ***Note:*** The case-study images were captured in a vertical camera orientation and subsequently rotated for horizontal web display. This camera orientation should not be confused with the lateral anatomical orientation of the specimen shown in Figure 2.
    ***Note:*** If all images in a folder share the same incorrect orientation, batch processing can be applied in Adobe Photoshop using Actions (File > Automate > Batch) to automatically rotate all images in the selected directory.
  c. Crop each image using a predefined cropping rectangle with fixed dimensions.
    **CRITICAL:** Because the specimen card dimensions are not proportional to the camera sensor dimensions, the captured images contain excess background area. Using a standardized cropping frame ensures that all images have identical dimensions and framing, which is necessary for consistent visualization and integration into the digital archive.
  d. Before specimen imaging, acquire a calibration reference image of a blank card containing a metric scale and, where available, a colour target, using the same camera position and settings as the specimen images.
    **CRITICAL:** The camera-to-specimen distance must remain constant throughout the entire imaging session to ensure accurate scale calibration.
  e. Process and verify the calibration image before batch acquisition, applying the same rotation, cropping, and export procedures intended for specimen images.
  f. Create a scale bar macro using the processed calibration image and record a macro in Photoshop that reproduces a graphical scale bar corresponding to the photographed reference.
  g. Apply the macro to all specimen images within the selected folder to automatically insert the scale bar into each image.
    ***Note:*** Because the calibration card is photographed during every imaging session without changing the camera–specimen distance, the automatically applied scale bar accurately reflects the real dimensions of the specimens.

### Label transcription, taxonomic validation, and Darwin Core data structuring

**Timing: 4 min per specimen**

This section describes label transcription, taxonomic validation, image–metadata linkage, and automated generation of the digital collection interface.

6. Label transcription.
  a. Transcribe collector, locality, date, sex, identification, catalogue information, and other label content into the Darwin Core-based spreadsheet.
    ***Note:*** OCR or multimodal AI tools may be used to generate a preliminary transcription, particularly for printed labels, but every automatically extracted value must be verified against the image or original material.
  b. Verify transcription accuracy against the specimen image or original labels.
  c. Verify taxonomic consistency by checking spelling, authorship, genus-species combinations, taxon rank, species versus subspecies identification, synonyms, and obsolete combinations against an appropriate specialist taxonomic source.
  d. Preserve historical or label identifications separately when scientifically relevant, while using the accepted name for normalized taxon-level navigation.
7. Linking images and metadata.
  a. Run the Python script described in Box 1 (see supplemental information; Figure 3) to extract identifier-based image filenames and associate them automatically with the corresponding spreadsheet records. Stable identifiers, rather than taxon-folder names, are used because determinations can change.

**Figure 3:**
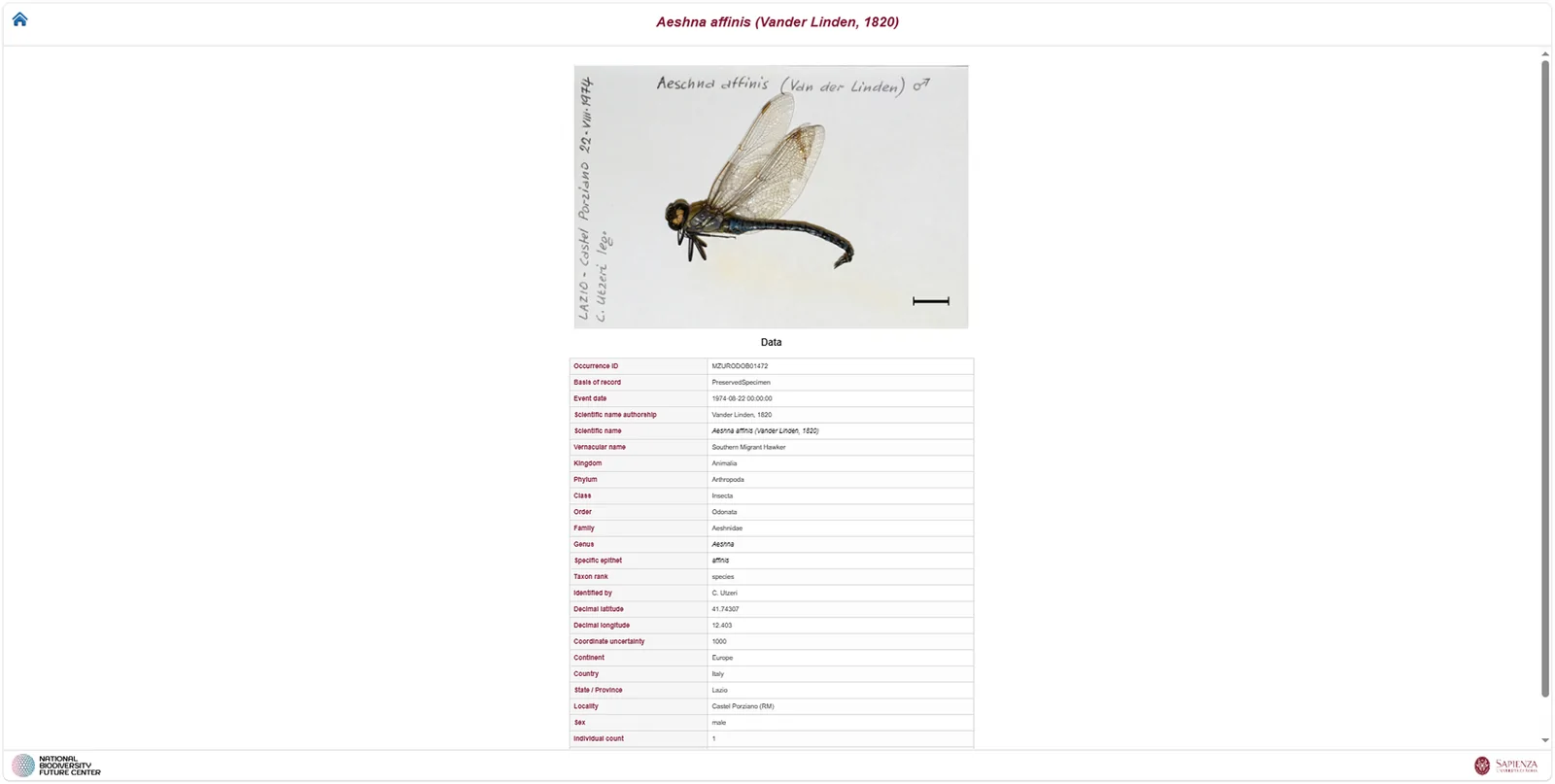
Individual specimen page generated from the digital collection interface Screenshot of a single specimen page automatically generated by the Python script. The page displays the high-resolution image of the specimen alongside a table containing all relevant metadata transcribed from the original specimen label. Metadata fields include collector, locality, date, identifier, and other associated information. All data are dynamically linked from the Excel dataset, ensuring accurate correspondence between the specimen image and its associated records. The scale bar visible in the specimen photograph is part of the original specimen image displayed on the web page.
  b. Confirm the correct pairing among occurrenceID, catalogNumber, specimen metadata, and every associated image before generating the web archive.
8. Automated generation of the digital collection interface.
  a. Before generation, ensure that accepted names and taxonomic ranks have been validated so that spelling variants, synonyms, and obsolete combinations do not create duplicate taxon pages.
  b. Execute the Python scripts described in Boxes 2–6 (see supplemental information; Figures 4, 5, 6, 7, 8, 9, 10, 11, and 12) to generate interconnected specimen pages, normalized taxon-summary pages, and multi-field search interfaces.

**Figure 4:**
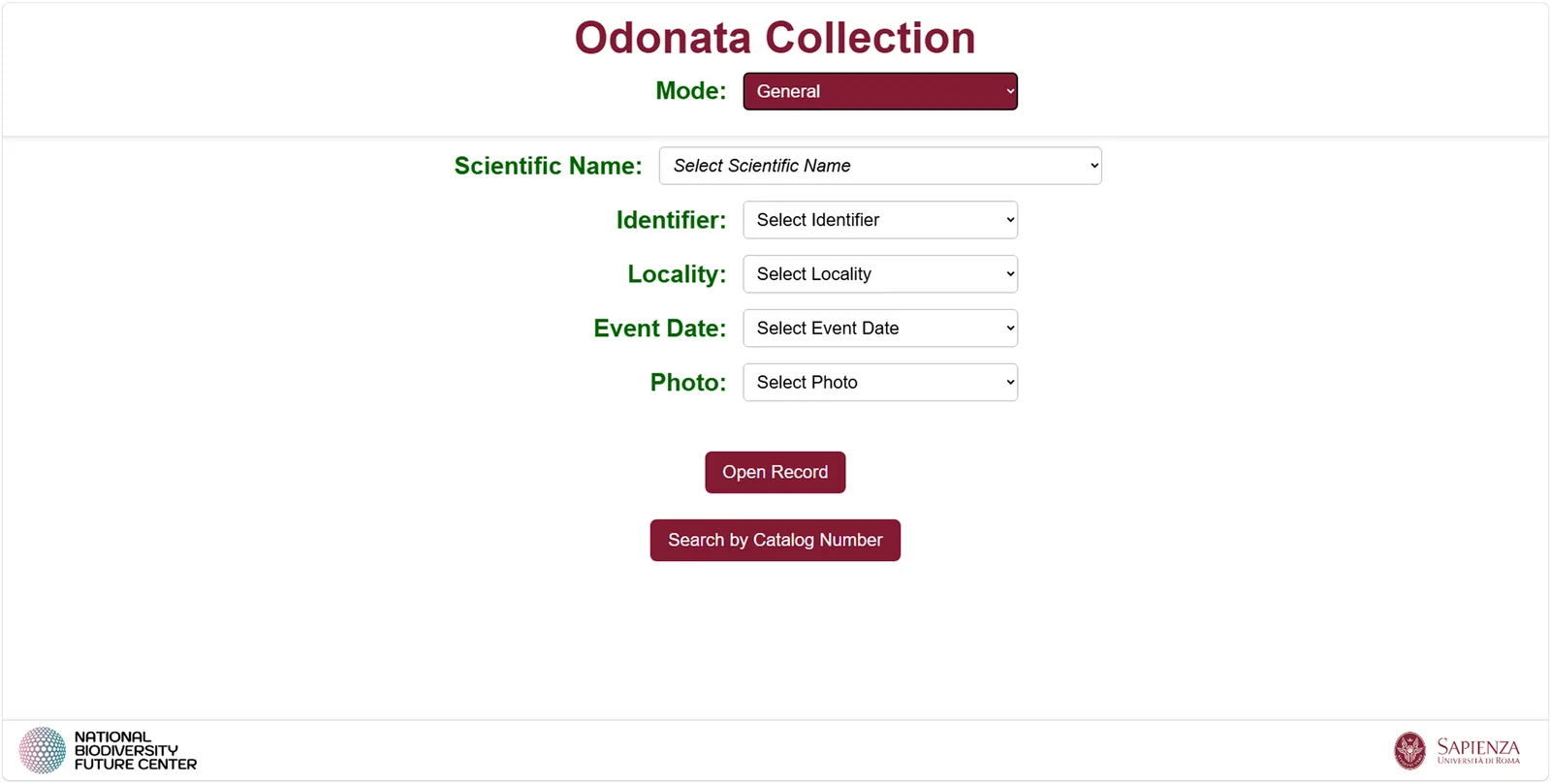
Home page of the automatically generated digital collection interface The General mode supports multi-field exploration using dynamically populated values derived from the curated dataset, including scientific name, identifier, locality, date, and image. Additional modes provide taxon-based and identifier-based access. Taxon navigation uses normalized accepted names, while complete specimen metadata and historical identifications remain available in the corresponding occurrence pages.

**Figure 5:**
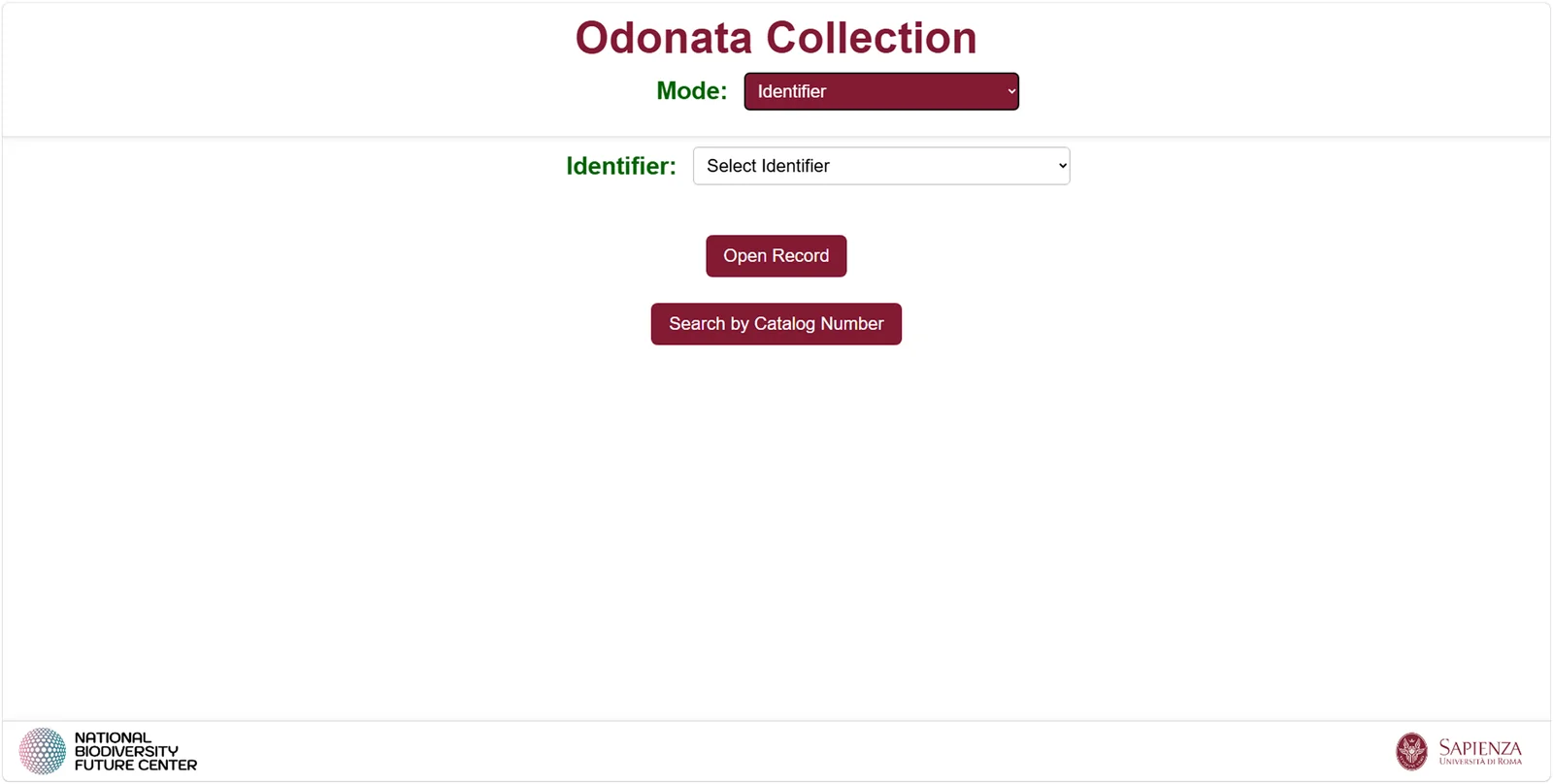
Specimen search by Identifier Screenshot of the specimen search interface accessed from the Home page by selecting the Identifier search mode. Users can select one or more specimen identifiers from the dropdown menu to locate the corresponding specimen page, which displays the image and associated metadata.

**Figure 6:**
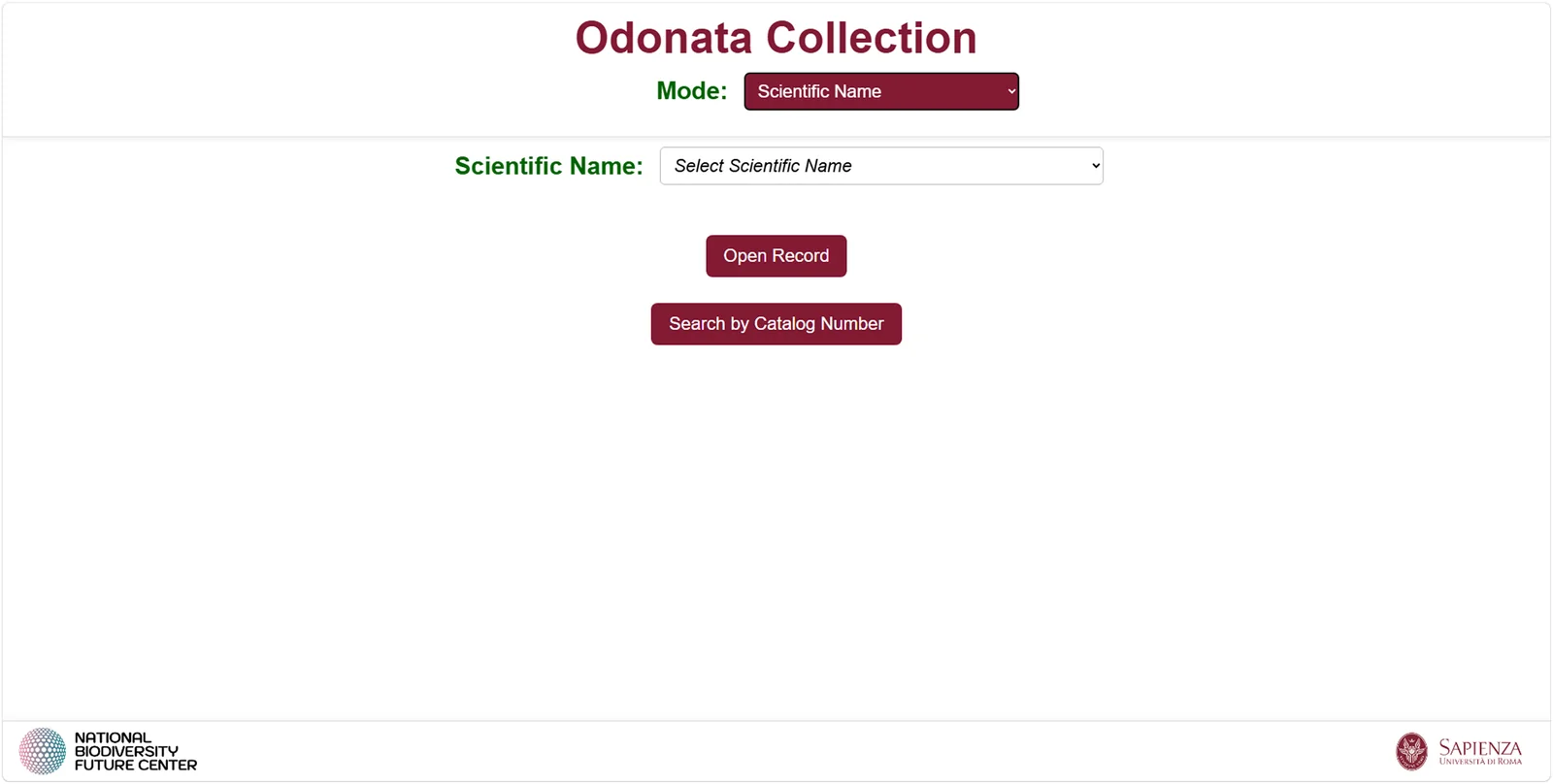
Specimen search by Scientific name Screenshot of the specimen search interface accessed from the Home page by selecting the Scientific name search mode. Users can select a species from the dropdown menu to access a summary page listing all specimens of that species, with links to individual specimen pages showing images and metadata.

**Figure 7:**
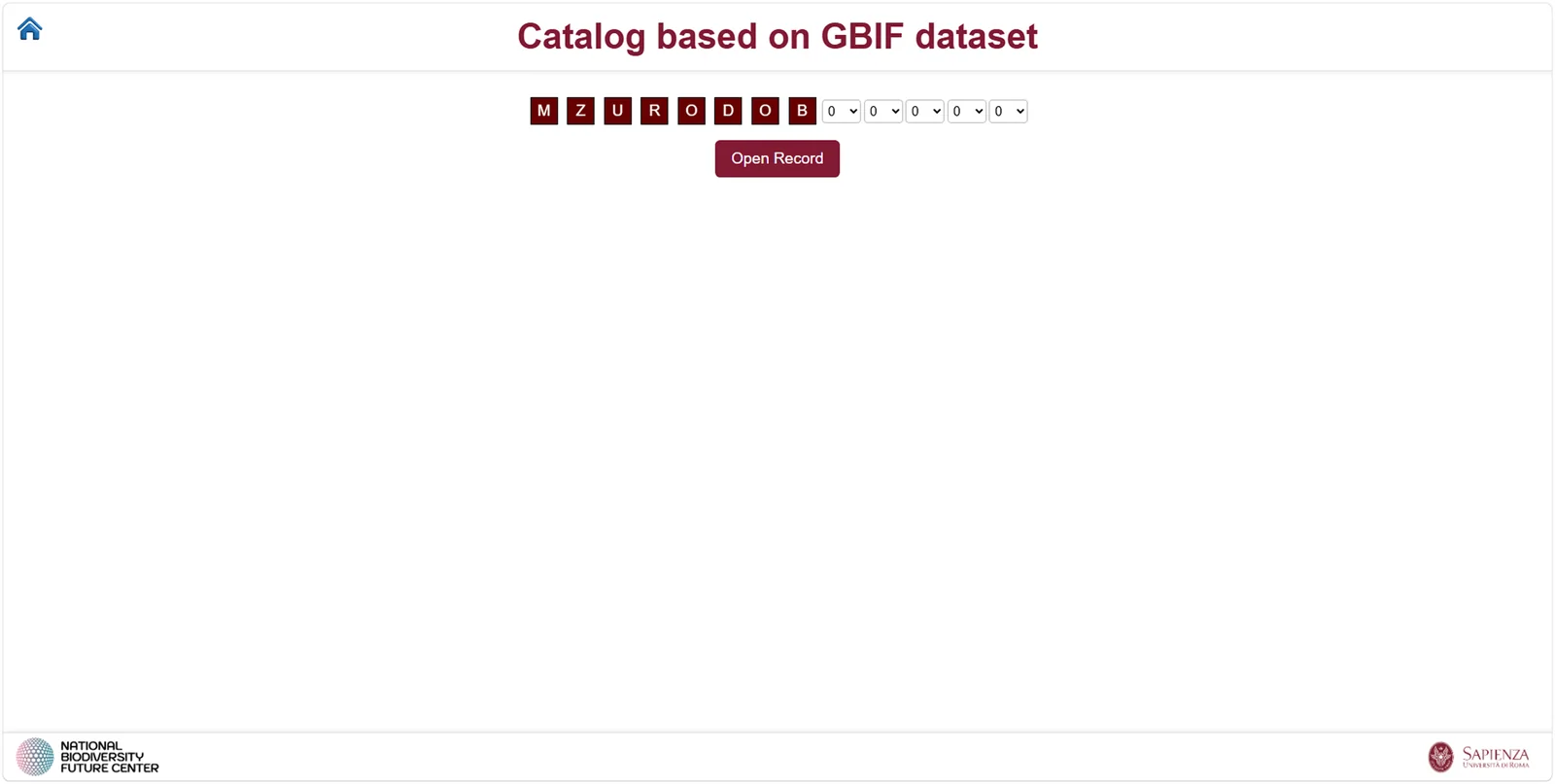
GBIF catalog access from the Home page Screenshot of the interface accessed by selecting the Go to GBIF catalog option on the Home page. This function allows users to reconstruct GBIF-compliant specimen codes directly from the dataset and navigate to the corresponding specimen images and metadata. The interface links Excel records to their digital representations, facilitating verification and integration of collection data with biodiversity data infrastructures.

**Figure 8:**
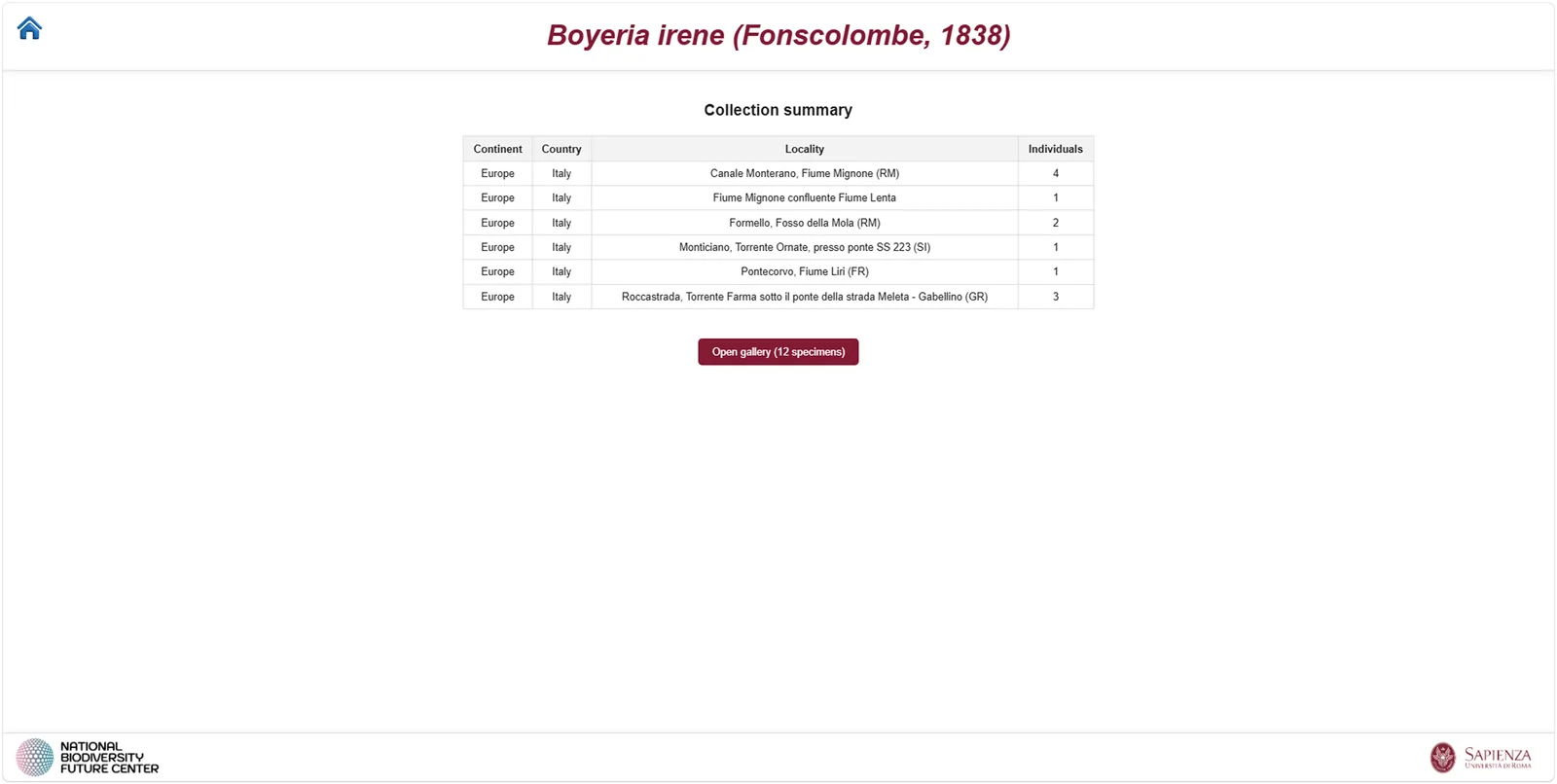
Species-level summary page generated by the Python script Screenshot of the HTML page automatically generated for a specific species. The page displays a summary table listing all collection localities for specimens of that species. At the bottom of the table, a Go to Gallery button allows users to access a thumbnail gallery of all associated specimen images, providing easy navigation from metadata to visual records.

**Figure 9:**
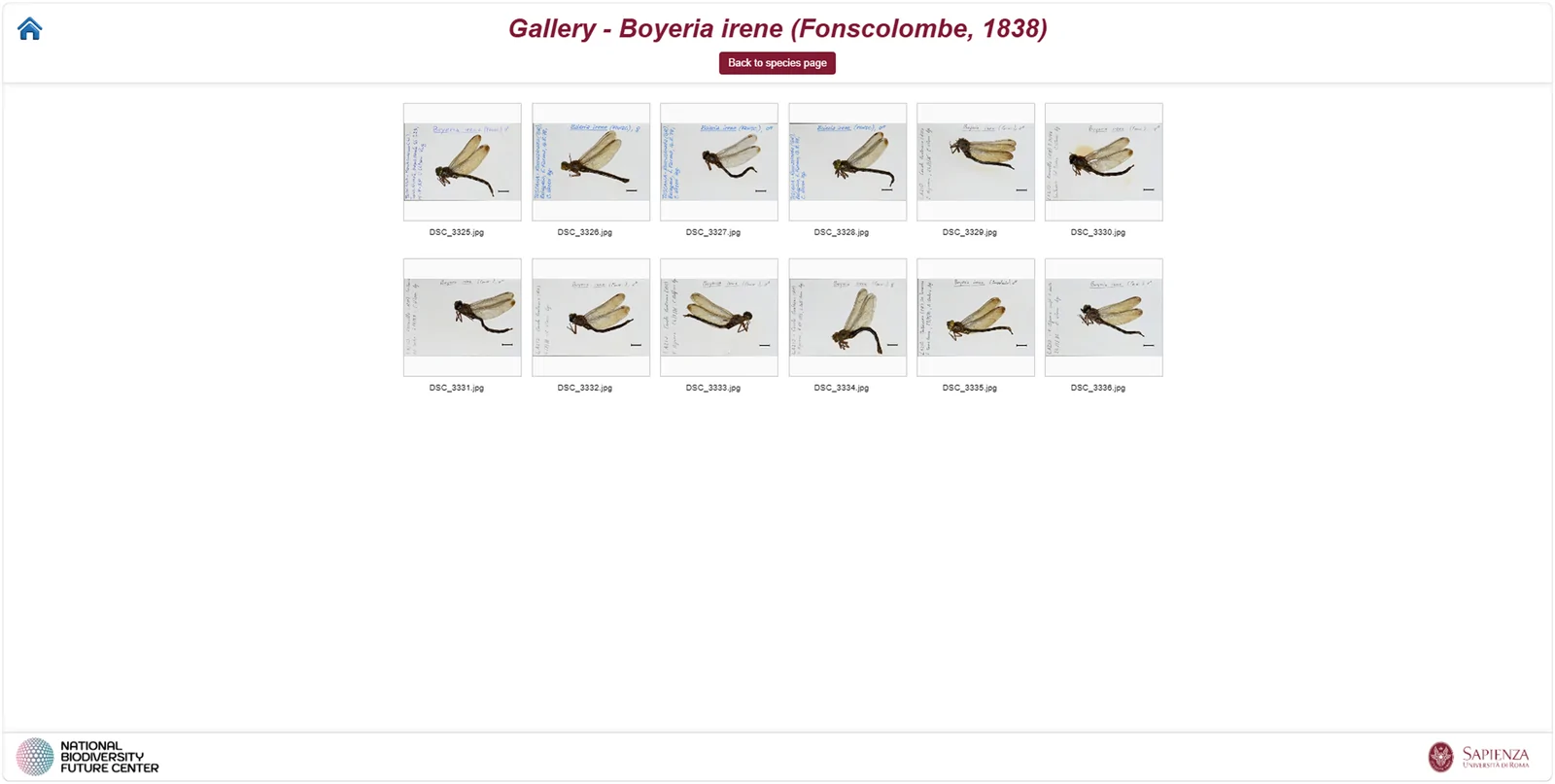
Species gallery page generated by the Python script Screenshot of the species-specific gallery page automatically generated from the dataset. The page displays thumbnail images of all specimens belonging to the selected species. Clicking on a thumbnail directs the user to the corresponding individual specimen page, showing the high-resolution image and associated metadata, allowing seamless navigation between visual and textual records.

**Figure 10:**
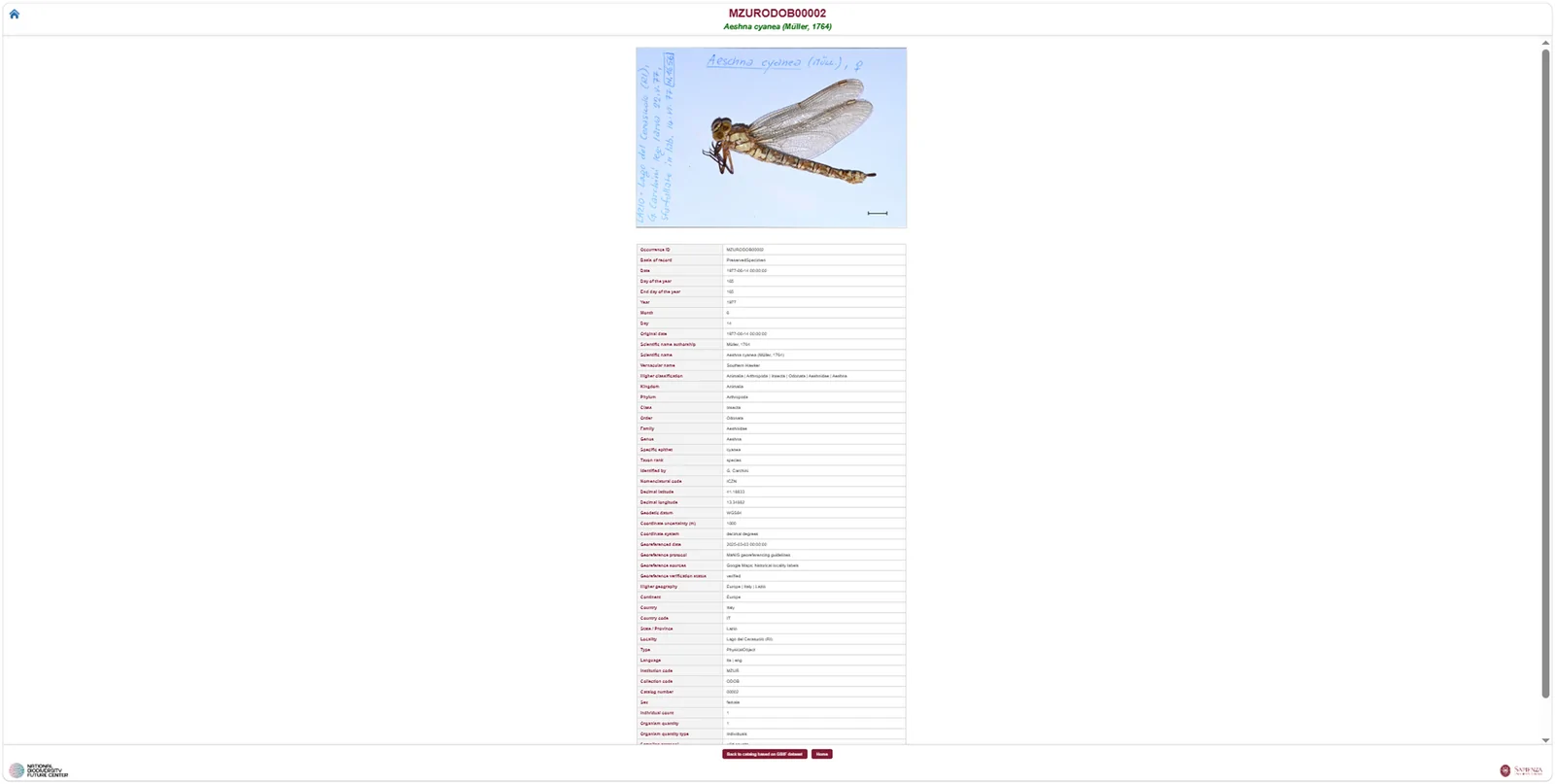
Individual specimen page accessed from the species gallery Screenshot of an individual specimen page opened by selecting a thumbnail from the species gallery. The page displays a high-resolution image of the selected specimen along with all associated metadata, including collector, locality, date, identifier, and other relevant information. This interface ensures accurate linkage between visual records and their corresponding dataset entries.

**Figure 11:**
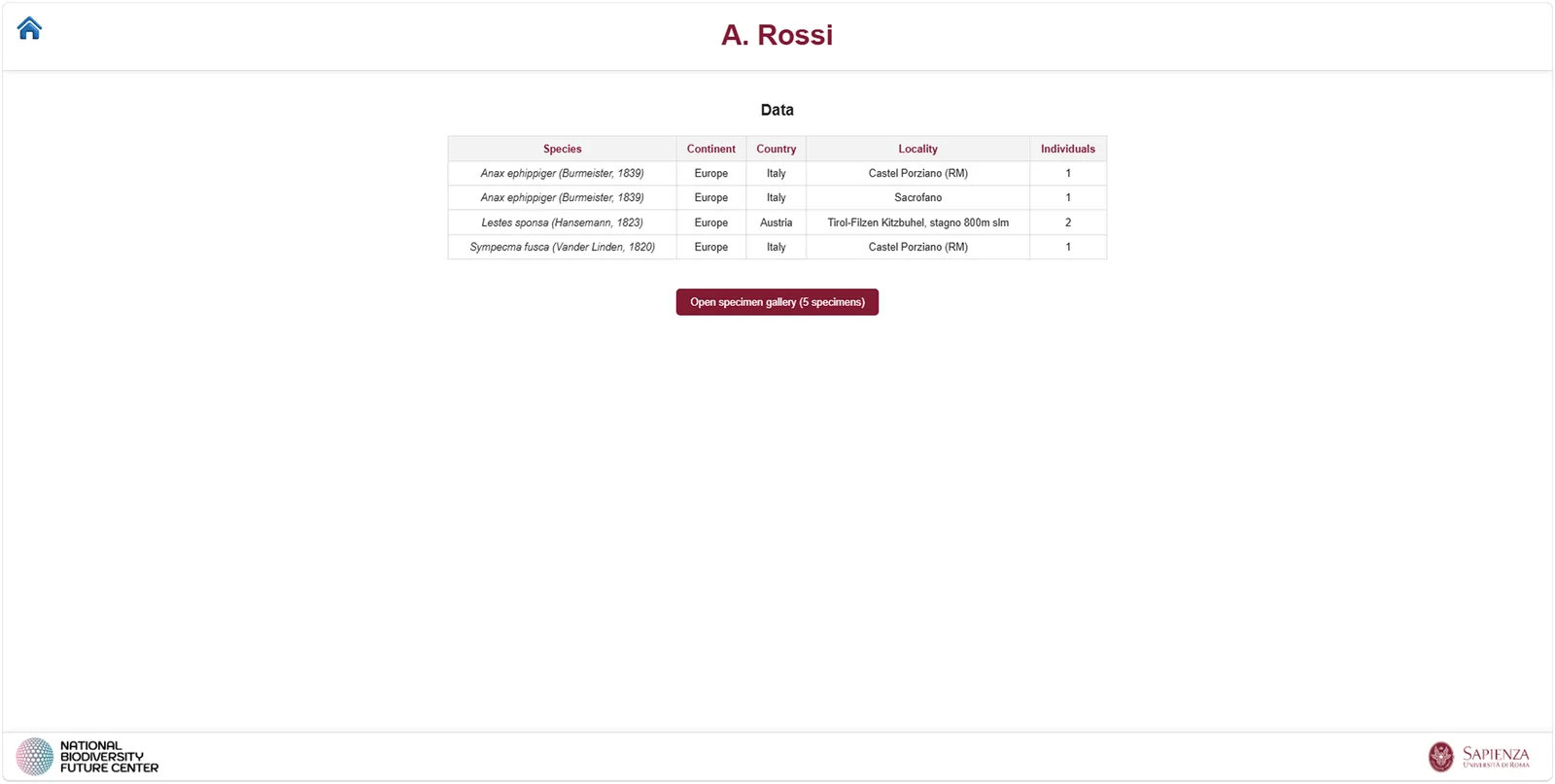
Identifier-based summary page generated by the Python script Screenshot of the HTML page automatically generated for a specific identifier. For each identifier present in the dataset, the page displays a summary table listing all species identified by that person and the corresponding collection localities. This interface allows users to quickly explore the contributions of individual identifiers and access the associated specimen images and metadata.

**Figure 12:**
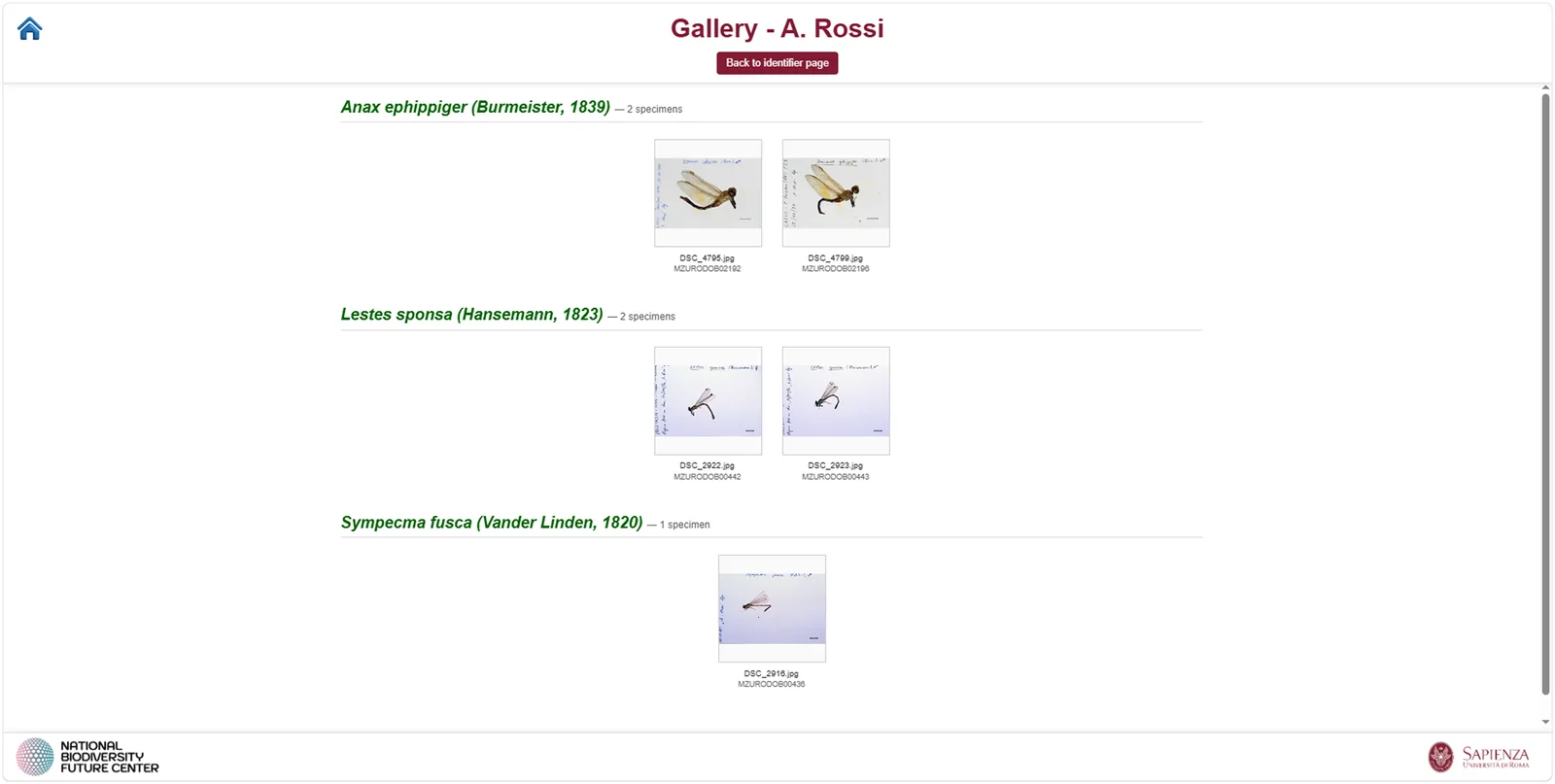
Identifier-based specimen gallery generated by the Python script Screenshot of the specimen gallery automatically generated for a specific identifier. The gallery displays the images of specimens associated with that identifier, organized by species, providing a visual overview of the corresponding records. Each image is linked to the relevant specimen page, allowing users to access the associated metadata and detailed specimen information.
  c. Share the generated HTML files, image directories, metadata tables, and supporting assets through a public GitHub repository or equivalent service.
    i. Preserve relative paths so that links among pages, images, and metadata remain functional.
    ii. Deploy the static interface through GitHub Pages without requiring a dedicated database server.
  d. Verify specimen pages, taxon summaries, multi-field search controls, external links, and image paths after deployment.

***Note:*** The interface should support retrieval not only by taxon, but also by fields such as identifier, locality, collecting date, catalogue number, and image.

***Note:*** A functional implementation of the complete workflow described in this protocol is publicly available through GitHub Pages at: Odonata Collection. The website provides direct access to the digitized Odonata collection and allows users to explore specimen-level pages, species galleries, identifier-based summaries, and the associated specimen images and metadata.

## Expected outcomes

The principal output of this protocol is a low-cost digital publication pipeline that converts curated specimen data and images into a searchable static website. Each occurrence is linked to one or more high-resolution images and an individual HTML page. Normalized taxon-summary pages aggregate all records assigned to the same accepted taxon, while the general interface supports multi-field consultation by taxon, identifier, locality, collecting date, catalogue number, and image. The complete Darwin Core-based dataset remains available for exchange and reuse, whereas the website displays a concise subset selected for readability.

When applied to the historical Odonata collection used as a case study, the workflow generates individual occurrence pages, normalized taxon-summary pages, image galleries, and interconnected search interfaces. The archive can be explored locally or deployed as a public website without a dedicated database server.

The workflow supports reliable linkage between specimen images and metadata, structured exploration of the collection, and reuse of the underlying occurrence data. Darwin Core terms support interoperability with biodiversity information systems, provided that identifiers, taxonomic names, geographic data, and field semantics are validated. GBIF may be used for occurrence publication and discovery, but specialist taxonomic resources should be used for authoritative name validation.

In particular, the dataset supports a wide range of research applications, including taxonomic revision, species distribution studies, biodiversity assessments, and the integration of historical occurrence records into macroecological, macroevolutionary, and conservation analyses. By converting museum collections into structured digital resources, the workflow facilitates the reuse of historical zoological data that are otherwise difficult to access or analyze at scale.

The protocol was validated through the complete digitization of the historical Odonata collection housed at the Zoological Museum of Sapienza University of Rome. The workflow processed 2,406 catalogued records, including specimen imaging, metadata transcription, image post-processing, and the generation of searchable HTML interfaces.

The resulting digital archive provides:

Standardized high-resolution specimen images suitable for documentation and morphological comparison, Darwin Core-structured metadata supporting exchange and integration with biodiversity information systems, individual occurrence-level HTML pages providing traceable access to images and metadata, normalized taxon-summary pages aggregating records under accepted names while preserving historical identifications where relevant, multi-field search interfaces supporting retrieval by taxon, identifier, locality, collecting date, catalogue number, and associated image, and an easy-to-read static web archive that can be shared through GitHub Pages or an equivalent hosting service.

The structured organization of the dataset ensures full traceability between images and metadata and improves data quality control during large-scale digitization. Overall, the resulting resource enhances accessibility and usability of museum collections for research in taxonomy, systematics, biogeography, and biodiversity informatics.

## Quantification and statistical analysis

The protocol generates a structured dataset containing specimen metadata and associated image files. Data processing and validation are performed through a combination of manual verification and automated computational procedures implemented in Python scripts.

Label information is transcribed into a spreadsheet structured using Darwin Core terms. AI-assisted transcription may be used as a preliminary aid, but both manual and automated transcriptions require verification. Taxonomic records are also checked for spelling, authorship, accepted usage, rank, synonymy, and consistency before taxon-level pages are generated.

The Python scripts process the curated spreadsheet to extract relevant metadata fields and generate specimen pages, normalized taxon summaries, and navigation interfaces. Records are associated with images through identifier-based filenames. Basic summaries include the number of specimens per accepted taxon, collector or identifier, locality, and collecting date; users can also retrieve records through multiple independent fields.

To ensure reproducibility of the workflow, the dataset used for testing the scripts can be provided as supplementary material together with the Python code used to generate the HTML archive. Researchers applying this protocol can therefore replicate the full analysis pipeline and verify the integrity of the generated outputs.

## Limitations

This protocol is optimized for dry entomological specimens stored in paper or card envelopes and their associated labels. The identifier-management, data-structuring, and HTML-generation components may be adapted to pinned, slide-mounted, or fluid-preserved specimens, but those preservation media require dedicated handling and imaging procedures.

Historical collections often include handwritten labels that may be faded, damaged, incomplete, abbreviated, or difficult to interpret. OCR and multimodal language models may assist preliminary transcription, but historical place names, handwriting, and specialist terminology still require expert verification. Manual transcription is time-intensive and may limit scalability in large collections. Both human and AI-assisted transcription can introduce errors; therefore, systematic verification against the image or original label is required, and a second-person review is recommended for ambiguous records.

Image quality depends on the imaging setup, including lighting conditions, specimen positioning, camera stability, and resolution. A minimum camera resolution of 20 megapixels is recommended to ensure readability of labels and preservation of morphological details. Inconsistent illumination or suboptimal positioning may reduce image quality and affect downstream verification.

Correct association between specimen images and metadata depends on stable identifier-based filenames and structured input directories. The workflow uses occurrenceID or the complete institutional specimen identifier as the filename prefix, rather than taxon names, because identifications may change over time. Automated validation should flag missing, duplicated, or unmatched files before page generation.

Despite these limitations, the protocol substantially improves the accessibility and usability of biodiversity data when applied consistently and with standardized procedures. The automated workflows reduce manual effort in metadata organization, HTML generation, and online publication of large specimen datasets.

Although specimen handling and label transcription still require manual intervention, the automated Python-based workflows substantially reduce repetitive processing time associated with metadata organization, HTML generation, and online publication of large specimen datasets.

## Troubleshooting

### Problem 1 (step 4)

Specimens appear blurred or out of focus in captured images.

### Potential solution

Verify camera focus settings before each imaging session and refocus whenever the specimen height or position changes. Use a fixed mounting stage to stabilize specimens and minimize movement during image capture. Capture a test image and confirm sharpness before proceeding with batch imaging.

### Problem 2 (step 6)

Incomplete or inconsistent transcription of specimen label data leads to missing metadata fields.

### Potential solution

Use a standardized metadata template (e.g., Darwin Core spreadsheet) with mandatory fields highlighted. Implement a double-check procedure where a second operator reviews transcribed data at the end of each imaging batch.

### Problem 3 (steps 4 and 5)

Image files are accidentally misplaced, overwritten, or lost during data transfer.

### Potential solution

When possible, use tethered capture to save images directly to the workstation with the stable specimen identifier as the filename prefix. Otherwise, transfer files after each imaging batch, verify file counts and checksums where feasible, and maintain automatic backups in at least two storage locations before deleting temporary copies.

### Problem 4 (steps 3, 6, and 7)

Large digitization projects often involve processing a high number of specimens in a single session. During imaging and data transcription, specimen labels may be incorrectly matched with image files or with the corresponding rows in the Darwin Core spreadsheet, leading to data inconsistencies and potential loss of record traceability. This problem is particularly frequent when multiple operators handle imaging and data entry simultaneously.

### Potential solution

Maintain a strict identifier-based workflow during imaging and transcription. Display or scan the stable specimen identifier in the first image and use it as the filename prefix. Do not write temporary image numbers on original historical labels; use a removable imaging slip, barcode, or the protective envelope when an additional temporary marker is required. Automated scripts should compare identifiers in filenames with occurrenceID values in the Darwin Core table and report missing, duplicated, or unmatched records.

In addition, automated tools can assist in verifying file alignment. For example, scripts that generate a list of image filenames from the selected directory can be compared with the order of records in the Darwin Core spreadsheet, enabling rapid detection of inconsistencies between images and metadata.

## Resource availability

### Lead contact

Further information and requests for resources and reagents should be directed to and will be fulfilled by the lead contact, Claudio Chimenti (claudio.chimenti@uniroma1.it).

### Technical contact

Technical questions on executing this protocol should be directed to and will be answered by the technical contact, Davide Tamagnini (davide.tamagnini@uniroma1.it).

### Materials availability

This study did not generate any new unique reagents.

### Data and code availability

• The Python scripts used in this protocol are publicly available in the GitHub repository: https://github.com/Sapienza-BBCD/Odonata-digitization-scripts. An archived version of the repository is deposited in Zenodo (https://doi.org/10.5281/zenodo.22249054), providing a permanent DOI for citation and long-term preservation of the code.
• A functional implementation of the workflow described in this protocol is publicly accessible through GitHub Pages at Odonata Collection. The website provides access to the digitized *Odonata* collection and demonstrates the outputs generated by the workflow, including specimen-level HTML pages, species-based galleries, identifier-based summaries, search interfaces, specimen images, and associated metadata.
• The datasets supporting this protocol consist of specimen metadata organized according to Darwin Core standards and example image files used for testing the workflow. Example input files used to validate the scripts are available within the GitHub repository. The published article includes all datasets and code necessary to reproduce the workflow described in this protocol.

## Acknowledgments

This project has been developed in the framework of the National Biodiversity Future Centre (NBFC), funded by the Italian Ministry of University and Research (PNRR, Missione 4 Componente 2, “Dalla ricerca all’ impresa,” Investimento 1.4, project CN00000033).

## Author contributions

C.C. established this protocol. C.C. wrote the initial draft and prepared all figures in this protocol. D.T. and C.C. revised subsequent drafts. C.C. oversaw project administration and provided materials and resources for this study. D.T. and C.C. granted final approval of the article.

## Declaration of interests

The authors declare no competing interests.

## Supplemental information

Document S1. Boxes S1–S6

Data S1. Complete Darwin Core-based occurrence dataset of 2,406 catalogued Odonata specimens from the Zoological Museum of Sapienza University of Rome, related to Steps 6–8
