# Supplementary material for: Protocol for low-cost photographic digitization and automated HTML publication of envelope-preserved entomological museum collections
Source: STAR Protoc. 2026 Sep 28;7(4):104871. doi: 10.1016/j.xpro.2026.104871 (PMC13639734; doi:10.1016/j.xpro.2026.104871)
Supplement: Supplementary file 1 [file mmc1.pdf]

Supplemental Information for "Protocol for low-cost photographic digitization and automated HTML publication of envelope-preserved entomological museum collections

[Davide Tamagnini,[1,2*] and Claudio Chimenti [1,3,]]**

1 Department of Biology and Biotechnologies "Charles Darwin", Sapienza University of Rome, Rome, 00185, Italy.

2 Technical contact

3 Lead contact

*Correspondence: davide.tamagnini@uniroma1.it

**Correspondence: claudio.chimenti@uniroma1.it

Box 1. Image filename listing script, related to Step 7.

Current script: https://github.com/Sapienza-BBCD/Odonata-digitization-scripts/blob/main/elenca_file_1.py

This utility generates a text file containing the filenames of all specimen images stored in a selected directory. The list can be used during data preparation to check image completeness and to facilitate the association of image files with the corresponding specimen records in the Darwin Core spreadsheet.

The script is particularly useful for detecting missing, duplicated, or unexpectedly named image files before the HTML-generation steps.

When executed, the script prompts the user to select:

- the folder containing the specimen image files;
- the destination location where the text file containing the filename list will be saved.

Box 2. Specimen-level HTML page generator, related to Step 8.

Current script: https://github.com/Sapienza-BBCD/Odonata-digitization-scripts/blob/main/Dragonfly_01a_VER_07_2026.py

This script generates one HTML page for each occurrence record in the Darwin Core Excel dataset. Specimen images are obtained directly from the associatedMedia field, and the user can choose which Darwin Core fields are displayed on the generated pages.

The generated specimen pages constitute the basic record-level units of the digital archive. Shared visual assets are copied to the output directory, whereas the specimen-image reference remains linked to the value stored in associatedMedia.

When executed, the script prompts the user to select:

- the Darwin Core Excel file;
- the folder containing the logo files;
- the destination folder for the generated HTML files;
- the Home icon;
- the Darwin Core fields to be displayed on the specimen pages, using the graphical field-selection dialog.

Note: The current version does not require the user to select a separate specimen-image folder because image references are read from associatedMedia.

Box 3. Home page and multi-field search interface generator, related to Step 8.

Current script: https://github.com/Sapienza-BBCD/Odonata-digitization-scripts/blob/main/Dragonfly_02_VER_07_2026.py

This script generates the main collection index page (index.html), which serves as the entry point to the digital archive.

The interface provides three search modes: a general cascading search, a scientific-name search, and an identifier search. In the general mode, the dropdown menus are populated dynamically from the Darwin Core dataset and filter records sequentially by scientific name, identifier (identifiedBy), locality, event date, and associated image. The selected image links to the corresponding specimen-level HTML page.

When executed, the script prompts the user to select:

- the Excel file containing the Darwin Core dataset;
- the folder containing the previously generated specimen HTML record files;
- the destination folder in which index.html will be saved;
- the first logo file;
- the second logo file.

Box 4. GBIF-style catalogue and occurrence-page generator, related to Step 8.

Current script: https://github.com/Sapienza-BBCD/Odonata-digitization-scripts/blob/main/Dragonfly_03_VER_07_2026.py

This script generates a GBIF-style catalogue interface (catalogo_gbif.html) together with one occurrence-level HTML page for each occurrenceID in the Darwin Core dataset.

Each catalogue code links directly to its corresponding occurrence page. The occurrence pages display the specimen image referenced in associatedMedia, the occurrenceID, the scientific name when available, and all non-empty Darwin Core metadata fields. The generated files are intended to reside in the same website root as index.html so that relative links remain compatible with GitHub Pages.

When executed, the script prompts the user to select:

- the Darwin Core Excel file;
- the website root/output folder in which the catalogue and occurrence pages will be generated;
- the Home icon;
- the two logo files used in the page template.

Box 5. Species-level summary and gallery generator, related to Step 8.

Current script: https://github.com/Sapienza-BBCD/Odonata-digitization-scripts/blob/main/Dragonfly_04_VER_07_2026.py

This script generates a species-level browsing structure from the Darwin Core dataset. For each scientificName, it creates a summary page and an associated gallery containing the specimen images belonging to that taxon.

The species pages summarize geographic information from the dataset, including continent, country, and locality, and link the gallery images to the corresponding numbered specimen pages. The script therefore provides taxon-based navigation while preserving direct access to the underlying specimen records.

When executed, the script prompts the user to select:

- the Excel file containing the Darwin Core dataset;
- the website root folder containing index.html and the numbered specimen pages;
- the Home icon;
- the left logo;
- the right logo.

Note: The website root selected here must be the same folder that contains index.html and the numbered specimen pages generated in the preceding workflow.

Box 6. Identifier-based summary page generator, related to Step 8.

Current script: https://github.com/Sapienza-BBCD/Odonata-digitization-scripts/blob/main/Dragonfly_05_VER_07_2026.py

This script reads the Darwin Core dataset, groups occurrence records by identifiedBy, and generates one HTML page for each identifier.

Each identifier page summarizes the species and locality information associated with that person's determinations. This interface allows users to explore the taxonomic contribution of individual identifiers and provides a structured route to the specimens associated with each determination.

When executed, the script prompts the user to select:

- the Excel file containing the Darwin Core dataset;
- the output folder in which the identifier pages will be saved;
- the Home icon;
- the left logo;
- the right logo.

Workflow note. The five HTML-generation scripts in Boxes 2–6 are designed to operate on the same curated Darwin Core dataset and to generate mutually connected static HTML resources that can be deployed through GitHub Pages or an equivalent static hosting service.
